# A randomized cross-over trial to determine the effect of a protein vs. carbohydrate preload on energy balance in ad libitum settings

**DOI:** 10.1186/s12937-019-0497-4

**Published:** 2019-11-09

**Authors:** Madeline J. Gibson, John A. Dawson, Nadeeja N. Wijayatunga, Bridget Ironuma, Idah Chatindiara, Fernando Ovalle, David B. Allison, Emily J. Dhurandhar

**Affiliations:** 1 School of Public Health Dean’s Office, University of Alabama at Birmingham, Birmingham, AL USA; 2 Department of Nutritional Sciences, Texas Tech University, Lubbock, TX USA; 3 Department of Kinesiology and Sports Management, Texas Tech University, Lubbock, TX USA; 4 University of Mississippi, Department of Nutrition & Hospitality Management, Oxford, MS USA; 5 School of Sport, Exercise and Nutrition, Massey University College of Health, Auckland, New Zealand; 6 Department of Medicine, University of Alabama at Birmingham School of Medicine, Birmingham, AL USA; 7 School of Public Health Dean’s Office, Indiana University, Bloomington, IN USA

**Keywords:** Protein, Egg, Preload, Energy balance, Macronutrients, Randomized trial, Cross-over trial

## Abstract

**Background:**

Although high protein diets have been tested in controlled environments for applications to weight management, it is not understood if adding high protein foods to the diet would impact ad libitum energy balance in the absence of other lifestyle changes.

**Methods:**

This double-blinded randomized crossover trial compared the effects of a protein shake (PS) to a carbohydrate shake (CS), consumed prior to each major meal to equate to 20% of total energy needs over the course of the day, on energy balance over two 5-day treatment periods in healthy adults with BMI 20–30 kg/m2. Tri-axial accelerometers estimated physical activity energy expenditure. Ad libitum energy intake was measured in a laboratory kitchen.

**Results:**

Energy balance was positive during both treatment periods but was not different between periods. There were no interactions between treatment and preload caloric dose or treatment and BMI status on energy balance. Satiety ratings did not differ for any pairwise comparisons between treatment and caloric dose. Controlling for gender and basal metabolic rate, thermic effect of food was greater for PS than CS.

**Conclusions:**

Preload periods significantly altered the macronutrient composition of the overall diet. This study found limited evidence that carbohydrate or protein preloads have differential effects on energy balance in short-term ad libitum settings.

**Trial registration:**

This trial was pre-registered on clinicaltrials.gov as NCT02613065 on 11/30/2015.

## Background

The causative factors contributing to positive energy balance sufficient to produce weight gain are complex and not well understood. Therefore, it is challenging to develop practical weight gain prevention strategies that are effective in ad libitum conditions, where many interacting environmental, social, and physiological factors are hypothesized to influence energy balance [1]. Only a few randomized trials have tested whether eating a specific food influences energy balance and propensity to gain weight in ad libitum settings [2]. Moreover, many interventions examine energy intake for one meal or 1 day only, and those results may not translate to weight changes over time.

Because dietary protein has been shown to influence satiety [3] and energy expenditure [4], it may exert some level of control over energy intake and energy balance in ad libitum settings. Amino acids in circulation and the GI tract act directly and indirectly, respectively, to influence central nervous system control of energy balance [5, 6]. High protein diets are thought to increase overall energy expenditure because the thermic effect of food is higher for protein (20–30% ingested energy) than carbohydrate and fat (5–10% and 0–3% of ingested energy, respectively) [7]. The protein leverage hypothesis proposes that protein intake is a significant driver of total food intake, such that food low in protein or essential amino acids increases intake, while food in high protein decreases energy intake [8]. This phenomenon has been documented in mice [9]. In humans, the spontaneous reduction in energy intake caused by forced high protein diets [10, 11] is significant enough to produce negative energy balance and weight loss [11]. On the other hand, lowering the percent of protein in the diet from 15 to 10% results in higher total energy intake [12]. Furthermore, recent evidence suggests that high intakes of protein may increase energy expenditure during weight loss maintenance [13]. Hence, if protein is consumed in high amounts it may reduce the onset of obesity or even be beneficial for its treatment [5]. However, practical strategies to meaningfully and consistently alter the macronutrient composition of the diet in ad libitum settings, in the absence of a set menu or highly restricted food access, have not been tested previously, and realistic ways to utilize protein for weight management are currently absent.

There is also very little evidence on how or if macronutrient intake is regulated in humans. One strategy to study this is to specifically prescribe macronutrient intake and examine ad libitum food choice and compensatory responses to each macronutrient. The results of two studies [14, 15] that have examined this are mixed.

Furthermore, protein’s influence on ad libitum satiety and energy balance may differ by protein source [14], and therefore by amino acid composition or protein quality [16]. The effect of protein on satiety is directly related to protein quality [16]. High quality protein sources have greater digestibility and essential amino acid content. In particular, the essential amino acid leucine has been shown to regulate energy intake through mTOR and AMPK signaling in the hypothalamus [5]. One such naturally-occurring protein rich in leucine and containing all essential amino acids is eggs [17]. Served as breakfast, eggs are more satiating than cereal [18] and more effective than bagels at inducing weight loss among individuals attempting to lose weight [19]. This suggests that eggs may be a useful food for controlling energy balance in ad libitum settings; yet, the effect of egg protein, specifically, under these conditions has not been examined. Previous single-meal studies have found that egg albumin may not be as effective at inducing satiety as other protein sources [20, 21], but this has not been examined over several days. Therefore, we conducted a short-term intervention to inform the need for a longer trial.

The primary objective of this study was to compare the effect of an egg protein preload to the effect of a carbohydrate preload consumed prior to each major meal on energy balance over two 5-day treatment periods. We hypothesized that energy balance would be favorable (either neutral or negative energy balance) during protein supplementation despite an ad libitum, buffet-feeding paradigm and whereas energy balance would be positive during maltodextrin supplementation. We examined satiety as a secondary outcome and hypothesized that satiety would be greater during the protein supplementation period compared to the maltodextrin supplementation period. Secondary analyses also examined whether the macronutrient content of the preloads affected subsequent ad libitum protein and carbohydrate intake, respectively, as well as the percent of the macronutrient’s contribution to total daily caloric intake. We hypothesized, based on the protein-leverage hypothesis, that supplementation of protein would reduce ad libitum protein intake, but that supplementation of maltodextrin would not influence ad libitum carbohydrate intake.

## Methods

The Institutional Review Boards of the University of Alabama at Birmingham (141121006) and Texas Tech University (505571) approved this study. This trial was pre-registered on clinicaltrials.gov as NCT02613065. The reporting of this article complies with the Consolidated Standards of Reporting Trials (CONSORT) guidelines (see Additional file 2).

### Participants

Participants were recruited from the Birmingham, AL metro area. The study population was males and females 30–50 years old with a body mass index (BMI) of 20–30 kg/m2. Interested persons were screened over the phone and, if eligible, invited for an in-person screen. The in-person screen included staff-measured height and weight, a urine pregnancy test, and completion of the Brief Symptom Inventory [22] and EAT-26 [23] questionnaires to measure psychological distress and eating disorder symptoms, respectively. Participants then provided written informed consent.

Participants were excluded for self-reported high levels of physical activity, major illness, smoking, statin use, use of an unstable dose of anxiety, depression, or steroid medications, food allergies or restrictions, claustrophobia, drug or alcohol abuse, participation in a weight loss program or special diet in previous 3 months, weight change > 5% in previous 6 months, use of medication that affects appetite, prior surgical procedure for weight control, EAT-26 score ≥ 20, BSI score ≥ 90th percentile, pregnancy, anticipating pregnancy, an unwillingness to take contraceptive measures, and nursing.

### Study design

**Fig. 1:**
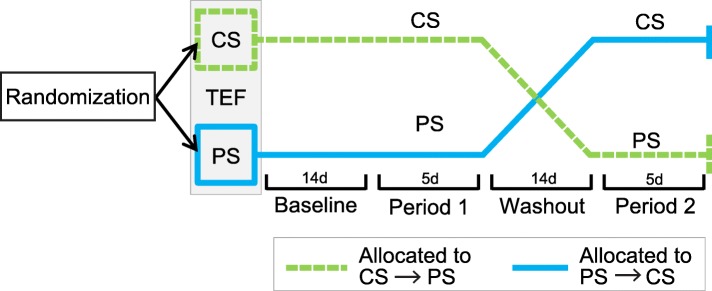
Study design from randomization through intervention completion. CS, carbohydrate shake; PS, protein shake; TEF, thermic effect of food

**Fig. 2:**
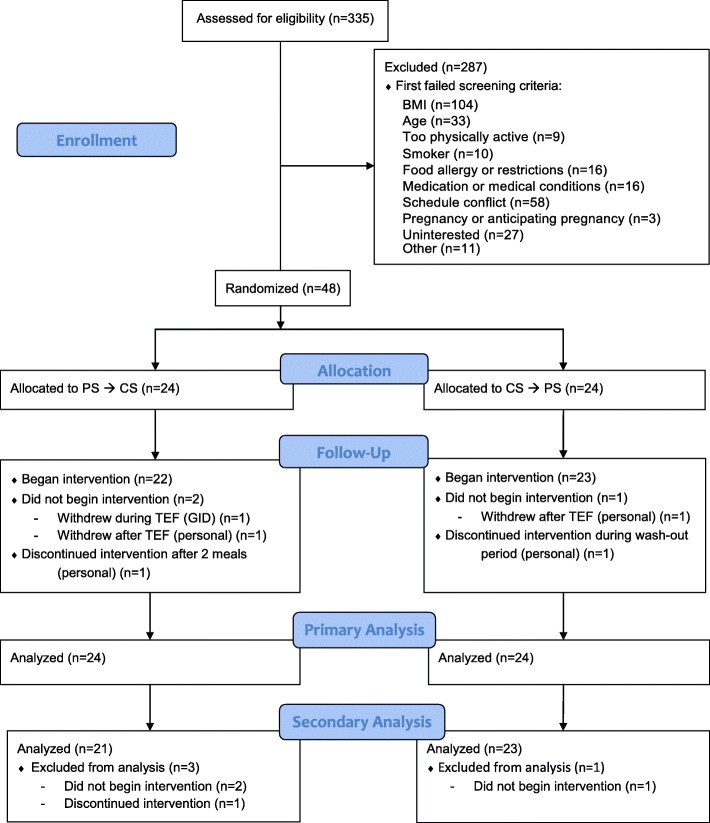
CONSORT flow diagram from enrollment through analysis. CS, carbohydrate shake; GID, Gastrointestinal discomfort; PS, protein shake; TEF, thermic effect of food

This double-blind randomized crossover trial sought to compare energy balance between two 5-day (Monday- Friday) treatment periods with a 2-week wash out between treatments (see Fig. 1). During each treatment period, participants ate breakfast, lunch and dinner (henceforth called major meals) at UAB’s Bionutrition Unit. Participants lived in their normal environments and came and went from the Bionutrition Unit for their major meals. Participants were asked to arrive at the Bionutrition Unit for each major meal between 6:45–8:30 am, 11 am – 12:30 pm and 4–5:30 pm, respectively. Participants often chose to sit together during mealtimes, but independent vs. group eating was not prescribed or recorded. Participants were required to finish either an egg white protein shake (PS) or a maltodextrin carbohydrate shake (CS) prior to consuming a buffet-style major meal. No time restrictions were placed on shake consumption, the transition from shake to buffet, or buffet consumption. Although buffets are known to cause greater food intake than meals with less variety [24], we wanted to test the practicability of the treatment in a quasi-real-world setting. Participants consumed the same shake for the entirety of each treatment period and were randomly assigned the order in which they received PS and CS (*n* = 48 randomized, see Fig. 2). We hypothesized that weight status may confer differing degrees of appetite regulation and therefore randomization was stratified by BMI (normal weight versus overweight). Allocation within each stratum was determined by block randomization with a block size of 4. The randomization scheme was created by the study statistician and provided to the study coordinator, who enrolled participants and assigned them to treatment allocation.

To strengthen the blinding process and minimize bias, participants and data collectors were unaware of the true study hypothesis and were told the purpose of the study was to determine the effects of a high-fiber and low-fiber shake on mood, which was self-reported by questionnaire on the first, third, and fifth day of each treatment period. The primary purpose of the questionnaire was not to measure mood per se but to increase the believability of the study rationale provided to participants. Participants were debriefed about the true study hypothesis at the end of their participation in the study. At that time, participants were also asked about any GI discomfort experienced during their participation, since the first participant withdrew for this reason.

### Basal metabolic rate, resting metabolic rate, and thermic effect of food

Basal metabolic rate (BMR), resting metabolic rate (RMR) and thermic effect of food (TEF) were assessed by indirect calorimetry [25] within 14 days prior to the first treatment period. Briefly, BMR was measured between 7 and 9 am, after a 12 h fast. Afterwards, participants consumed 300kcals of PS or CS, whichever they were assigned to for the first treatment period, and underwent RMR measurements for 10 min every 30 min for 6 h [25]. The first participant consumed 500kcals of PS; thereafter, the recipe was reduced to 300kcals to ease consumption during the allotted 10-min period. The 300 kcal dose, which was larger than what was given before each major meal, was chosen to illicit a measurable response according to standard TEF protocols. Participants were instructed to rest, but remain awake, in a reclined position during these 6-h. TEF was calculated as the area under the curve for energy expenditure, adjusting for baseline BMR.

### Preload shakes

Baseline BMR determined the caloric dose of each participant’s shakes. Shake dose was given as a percent of energy needs because energy balance was the primary outcome of the trial. Giving a large dose of supplement to someone with low energy needs would therefore bias energy intake positively relative to a person’s needs, or vice versa, and we wanted to eliminate this bias. In addition, meals where at least 20% of energy is derived from protein induces greater acute satiety after the meal compared to meals with less than 20% energy from protein [26]. Therefore, providing a protein preload with approximately 20% of energy needs over the course of the day should theoretically ensure that total daily energy consumed is at least 20% protein. This dose would likely also reflect what someone might take naturally if they were to consume the supplement on their own, outside a clinical setting or strict recommendation.

Participants were categorized into three BMR groups to minimize error when making the shakes: ≤1199 kcals/day, 1200–1599 kcals/day and ≥ 1600 kcals/day. Participants were assigned a 93 kcal, 130 kcal or 168 kcal dose, respectively, so that preload dose was relative to overall energy needs. For each BMR range, we multiplied the median value of the range by a physical activity level of 1.4. Twenty percent of this calculated value was provided through the preload shake at each meal. For the 93 kcal dose (henceforth called “low dose”), this translated to 18.6 g of protein for PS and 23.4 g of carbohydrate for CS. For the 130 kcal dose (henceforth called “medium dose”), this translated to 26 g of protein for PS and 32.8 g of carbohydrate for CS. For the 168 kcal dose (henceforth called “high dose”), this translated to 33.6 g of protein for PS and 42.4 g of carbohydrate for CS. Caloric doses and total shake volume were held constant across treatments. Shakes were made from water, Crystal Light no-calorie sweetener, and either NOW® Eggwhite Protein (PS) or NOW® Carbo Gain (CS). Energy density (kcal/g) was 4 kcal/g for PS and 3.73 kcal/g for CS. Bionutrition Unit staff developed shake recipes and experimented with dose of crystal light flavoring to sensorially match the shakes as much as possible. Crystal Light flavor (chosen by participant) and dose were also constant across treatments and were served in opaque containers. If participants detected differences between shakes, the differences were likely attributed to differences in fiber content, as they were told that shakes would have high and low fiber content. Shake recipes are provided in Table 1.

**Table 1:** Shake Recipes

BMR	Egg White Protein Shake		Maltodextrin Shake	
	Amount	Macronutrient composition (%)	Amount	Macronutrient composition (%)
800–1199				
Energy (kcal)	93	–	93	–
Component		–		
Water (ml)	120		120	–
Powder (g)	23.3		24.9	
Crystal light (g)	1.7		1.7	
Energy density (kcal/g)	4	–	3.7	–
Carbohydrate (g)	2.3	10.0	23.4	94.0
Protein (g)	18.6	80.0	0	0
Fat (g)	0	0	0	0
1200–1599				
Energy (kcal)	130	–	130	–
Component				–
Water (ml)	120	–	120	
Powder (g)	32.5		34.9	
Crystal light (g)	1.7		1.7	
Energy density (kcal/g)	4	–	3.7	–
Carbohydrate (g)	3.3	10.0	32.8	94.0
Protein (g)	26	80.0	0	0
Fat (g)	0	0	0	0
1600+				
Energy (kcal)	168	–	168	–
Component				–
Water (ml)	120	–	120	
Powder (g)	42		45.0	
Crystal light (g)	1.7		1.7	
Energy density (kcal/g)	4	–	3.7	–
Carbohydrate (g)	4.2	10.0	42.4	94.0
Protein (g)	33.6	80.0	0	0
Fat (g)	0	0	0	0

*BMR* Basal Metabolic Rate

### Energy intake

Major meals were served ad libitum, buffet-style in UAB’s Bionutrition Unit. The buffet provided mixed meals of “typical” American food in excess, to ensure that quantity did not limit intake. The macronutrient distribution of the buffet was 45–65% carbohydrate, 20–30% fat, and 10–35% protein, in keeping with USDA recommendations [27]. Several snack options were available to be taken between each major meal. Participants were asked to return snack packages, empty or otherwise, at the next major meal. Menus were designed by registered dietitians. Meals were prepared by trained staff who also weighed the food items before and after each meal or snack to obtain the total weight of foods consumed. After the first cohort (*n* = 5), the meal and snack menus were slightly adjusted to reduce food waste; nevertheless, the menus were consistent for each cohort. All cohorts after the first received the same meal and snack menu (see Additional file 1).

### Physical activity energy expenditure

During both treatment periods, participants were instructed to wear a tri-axial accelerometer (AntiGraph GT3X+, Pensacola, FL) over their right hip during waking hours. Accelerometers were distributed at breakfast on Monday and collected at dinner on Friday. Accelerations were summed over 1-min epochs and 60 min were used to determine non-wear time. Using the Freedson VM3 Combination algorithm [28] in Actilife v6.13.3, accelerometer data were processed to yield estimates of daily and weekly physical activity energy expenditure (PAEE) for each treatment period.

### Satiety and meal-liking

**Fig. 3:**
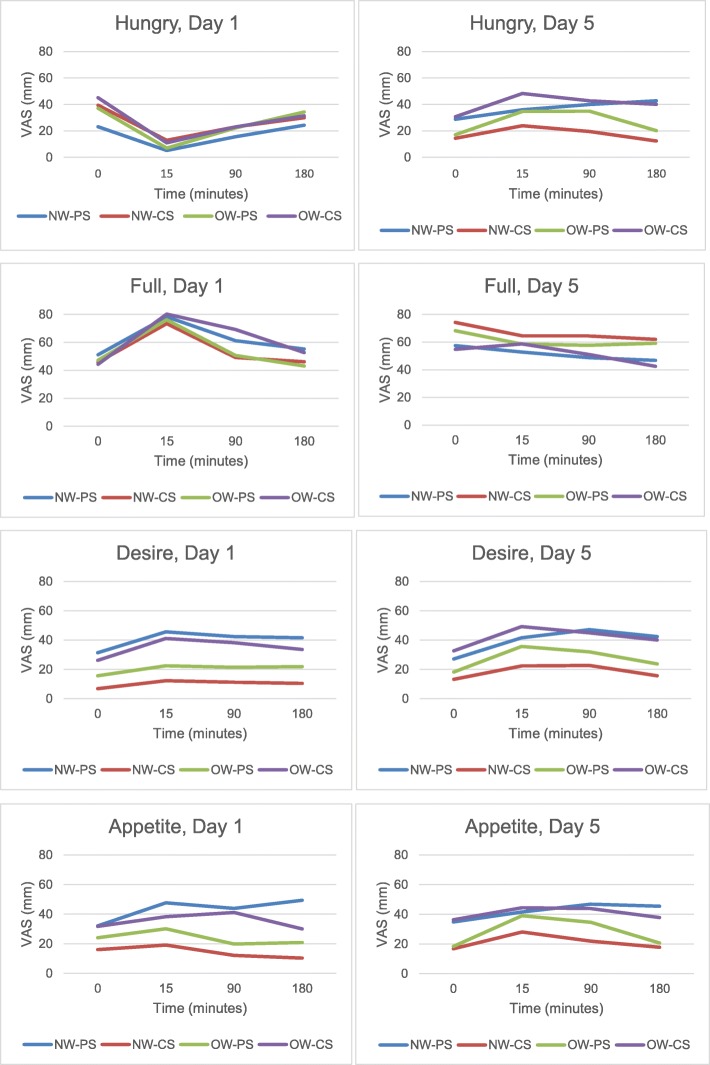
VAS satiety ratings reported by subjects immediately after shake consumption (0 min) and 15, 90, 180 min after buffet lunch completion on day 1 and day 5 of both treatment periods. NW-CS, participants with normal weight on carbohydrate shake (*n* = 11); NW-PS, participants with normal weight on protein shake (*n* = 9); OW-CS, participants with overweight on carbohydrate shake (*n* = 12); OW-PS, participants with overweight on carbohydrate shake (*n* = 12); Desire, self-reported VAS for “How strong is your desire to eat now?”; Full, self-reported VAS for “How full do you feel now?; Hungry, self-reported VAS for “How hungry do you feel now?”; Appetite, self-reported VAS for “How much food do you think you could eat now?”; VAS, Visual Analog Scale

A standard satiety questionnaire was administered immediately after shake consumption (0 min) and then 15, 90, and 180 min after buffet lunch completion on the first and fifth day of each treatment period. A 100 mm Visual analogue scale (VAS) anchored by “not at all” and “extremely” assessed fullness, hunger, ability to eat more and desire to eat more (see Fig. 3 legend for complete satiety questions). The 0 min satiety questionnaire was completed before partaking the buffet; the 15, 90, and 180 min post-meal questionnaire was completed elsewhere and returned to the Bionutrition Unit at the next meal. A second questionnaire was administered after every major meal to evaluate how much participants liked the shake and buffet meal. A nine-point Likert scale from “dislike extremely” to “like extremely” was used to answer the question, “How much did you like or dislike the meal you just had?”. Shake and buffet meal likings were evaluated together. All VAS ratings were recorded by pen and paper and subsequently scored and entered twice.

### Energy balance

Energy intake (EI) for each food item was calculated as follows: EI = total grams of the food item consumed * calories present in 1 g of that food item. For items that had a label, nutrition information was taken from the Nutrition Facts. For items without a label (e.g., fresh apples) the Nutrition Data System for Research was used. EI for each meal and snack were obtained by summing the calories of all food items consumed during each meal, including calories from the preload shake, and snack period, respectively. Calories from each day’s meals (*n* = 3) and snacks (*n* = 3) were summed for daily EI. Weekly EI was the sum of calories from all meals (*n* = 15) and snacks (*n* = 15) during the treatment period. Total daily energy expenditure was the sum of daily PAEE and BMR, while total weekly energy expenditure was the sum of daily PAEE and BMR*5. Energy Balance (EB) was calculated as the net difference between measured total daily energy intake and total daily energy expenditure per day over the treatment period.

### Statistical analysis

**Fig. 4:**
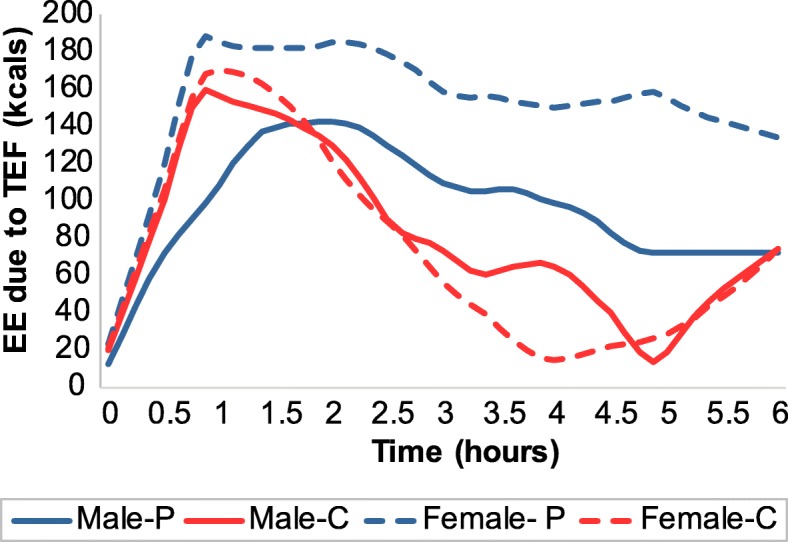
Energy expenditure due to thermogenesis of preload shakes, adjusted for baseline BMR and gender (*n* = 48). EE, energy expenditure; Female-CS, females on carbohydrate shake; Female-PS, females on protein shake; Male-CS, males on carbohydrate shake; Male-PS, males on protein shake; RMR, resting metabolic rate; TEF, thermic effect of food

Based on a previous study [25], our sample size (*n* = 48) had 80% power to detect a significant difference in energy balance between the two conditions at the 0.05 2-tailed alpha level. The crossover design provided power to detect treatment effects explaining as little as 8% of the variance in energy balance. Due to the crossover design, outcomes were assessed using linear mixed effects models, adjusting for caloric dose and including subject as a random effect. The primary analysis followed the intent-to-treat principle (*n* = 48). The secondary analysis included all subjects who completed the intervention (*n* = 43) and one subject who completed the first treatment period only (*n* = 44 total). Multiple imputation with 1000 imputations per analysis was used to account for missing data in accordance with the intent-to-treat principle [29]. While this is a high number of imputations, modern computation obviated any need to be frugal in this regard. Missing data in the secondary analyses were also treated with multiple imputation. The smoothed TEF curves of Fig. 4 are based on loess local polynomial regression [30]. The primary and secondary analyses were performed using R version 3.1.2 25, with specific use of the loess function and the lme4 package.

A post hoc analysis sought to determine if the macronutrient content of the preload (protein vs. carbohydrate) impacted the macronutrient content of the major meal and snack items consumed ad libitum. During data processing for this analysis, we made several assumptions: If the final weight of a food item or a beverage was missing, we assumed that the participant consumed all the food or beverage provided; If the serving weight was missing the average served weight was used; When the served weight for a beverage was missing, average weight was calculated based on 10 random selections of each beverage consumed by random participants and if 10 random selections were not found, the average of as many as were recorded was taken. If the type of beverage was missing, the average caloric value of all the beverages provided during the study was used. Along with the four participants who were excluded from the secondary analysis, the participant who did not attend the second treatment period was excluded from this analysis. Furthermore, we considered data for the last day of one participant as missing since the individual took unreasonably large amounts of food without recorded consumption amounts causing that data point to be an outlier. The difference in ad libitum carbohydrate and protein intake between the two treatment periods was calculated. Linear mixed models with repeated measures including treatment period, time, and caloric dose as fixed factors were used to study the differences in ad libitum protein and carbohydrate intake. Percent of daily calories from protein and carbohydrate was calculated as: (daily calories from macronutrient / total daily calories)*100. Student’s paired t-test compared treatment periods. This post hoc analysis was conducted using IBM SPSS software version 25 and Microsoft Excel.

## Results

Participant characteristics are described in Table 2. Briefly, 48 participants enrolled in the study in 2015 and 2016 and had a mean age of 39.5 ± 6.2 years and a mean BMI of 24.9 ± 2.7 kg/m^2^. Participants were predominantly female and non-Hispanic white. Forty-three participants completed the intervention (see Fig. 2 for CONSORT diagram). Four participants withdrew for personal reasons such as changes in availability. Two participants experienced gastrointestinal discomfort (GID) during the TEF measurement following PS consumption. One withdrew and one completed the study. Three additional participants reported GID during PS. During the debriefing interview, twelve participants reported GID during PS; six others reported GID without a time reference. None withdrew from the study, asked to have their data removed, or required medical attention. During data analysis, we learned that one participant may have had a BMI over 30. However, the BMI was calculated as under 30 during the in-person screen.

**Table 2:** Participant characteristics at baseline^a^

Gender	
Male	19 (39.6)
Female	29 (60.4)
Age, years	39.5 ± 6.2
Race	
Black	11 (22.9)
White	29 (60.4)
Asian	6 (12.5)
Hispanic	1 (2.1)
Pacific Islander	1 (2.1)
BMI (kg/m^2^)	24.9 ± 2.7
BMI < 25	24 (50.0)
BMI ≥ 25	24 (50.0)
Shake dose	
Low	9 (18.8)
Medium	29 (60.4)
High	10 (20.8)

^a^Data are expressed as frequency (%) or mean ± SD for *n* = 48

For the primary analysis (intent-to-treat, *n* = 48), EB was positive during both treatment periods but did not differ between periods (*p* = 0.70). EI and PAEE also did not differ between treatment periods (*p* = 0.87 and *p* = 0.62, respectively). There were no significant effects of preload caloric dose on EB or EI (all *p* > 0.088). There was a significant effect of dose on energy expenditure such that those receiving the high dose had higher overall energy expenditure than those receiving the low dose (*p* = 0.0013). This difference is not surprising and is likely due to larger body size and BMR in those receiving the high dose compared to those receiving the low dose. Energy expenditure did not differ between those receiving the low and medium doses (*p* = 0.92). There were no statistically significant interactions between treatment and preload caloric dose or treatment and BMI status on energy balance. Carryover effects were not significant and were dropped from the model.

Results from the secondary analysis (*n* = 44) mirrored those of the primary analysis except that there was a significant interaction between treatment and high dose on energy balance, such that participants receiving the high dose had + 882 kcal/week higher energy balances on CS than PS (*p* = 0.030). Values for TEE, PAEE, EI and EB from the secondary analysis are presented in Table 3; the standard error values include uncertainty arising from missing values, as quantified by multiple imputation.

**Table 3:** Total energy expenditure (kcal), physical activity energy expenditure (kcal), energy intake (kcal) and energy balance by weight status and treatment period^*^

	CS-N	PS-N	CS-O	PS-O	*P*-value
TEE	9459.8 ± 1953.2	9597.6 ± 2117.9	10716.1 ± 1679.5	10778.5 ± 1751.1	0.43
PAEE	1923.8 ± 910.7	2049.1 ± 1181.6	2444.2 ± 617.7	2480.6 ± 639.5	0.44
EI	12539.4 ± 3796.7	12072.8 ± 2996.1	12429.9 ± 2665.1	12730.1 ± 2517.8	0.13
EB	3079.6 ± 2727.8	2475.2 ± 2447.2	1713.8 ± 2422.5	1951.7 ± 2596.7	0.14

^*^Data are expressed as mean ± SE for *n* = 44. *P*-values are for the treatment*BMI status interaction, after adjusting for caloric dose. EB, Energy balance; EI, Energy intake; CS-N, subject with normal weight during CS; PS-N, subject with normal weight during PS; CS-O, subject with overweight during CS; PS-O, subject with overweight during PS; PAEE, Physical activity energy expenditure; TEE, Total energy expenditure

Figure 3 illustrates self-reported VAS satiety scores recorded at 0, 15, 90, and 180 min after preload consumption. Ratings of hunger, fullness, desire to eat, and ability to continue eating did not differ for any pairwise comparison between treatment and preload caloric dose (all *p* > 0.28). Controlling for gender and baseline BMR, TEF area under the curve was greater for PS than CS (*p* = 0.0037, see Fig. 4).

We conducted a reliability check for the self-reported meal-liking data, since the same meals were served on the first, second, third, etc. days of each treatment period. The check revealed high correlations between average daily meal-liking across treatment periods (rho = 0.72, mean = − 0.044 Likert units, median = 0 Likert units, Interquartile Range = 1.04 Likert units). As expected, there was also a weak but significant correlation between average daily meal-liking and daily EI (rho = 0.156, *p* = 0.0011).

A sensitivity analysis explored the role of self-reported gastrointestinal distress (GID) during PS on EI. Participants who reported GID on PS, had greater energy intake (138 kcals/day, *p* = 0.03) than participants who did not report GID. However, there was no interaction between GID and treatment dose (*p* = 0.55); participants reporting GID had greater EI than non-reporters during both treatment periods. Moreover, when comparing these two groups, average daily meal-liking did not differ between treatments (*p* = 0.058) or during PS specifically (*p* = 0.12). Therefore, there was no evidence that GID differentially impacted EI or meal-liking.

**Fig. 5:**
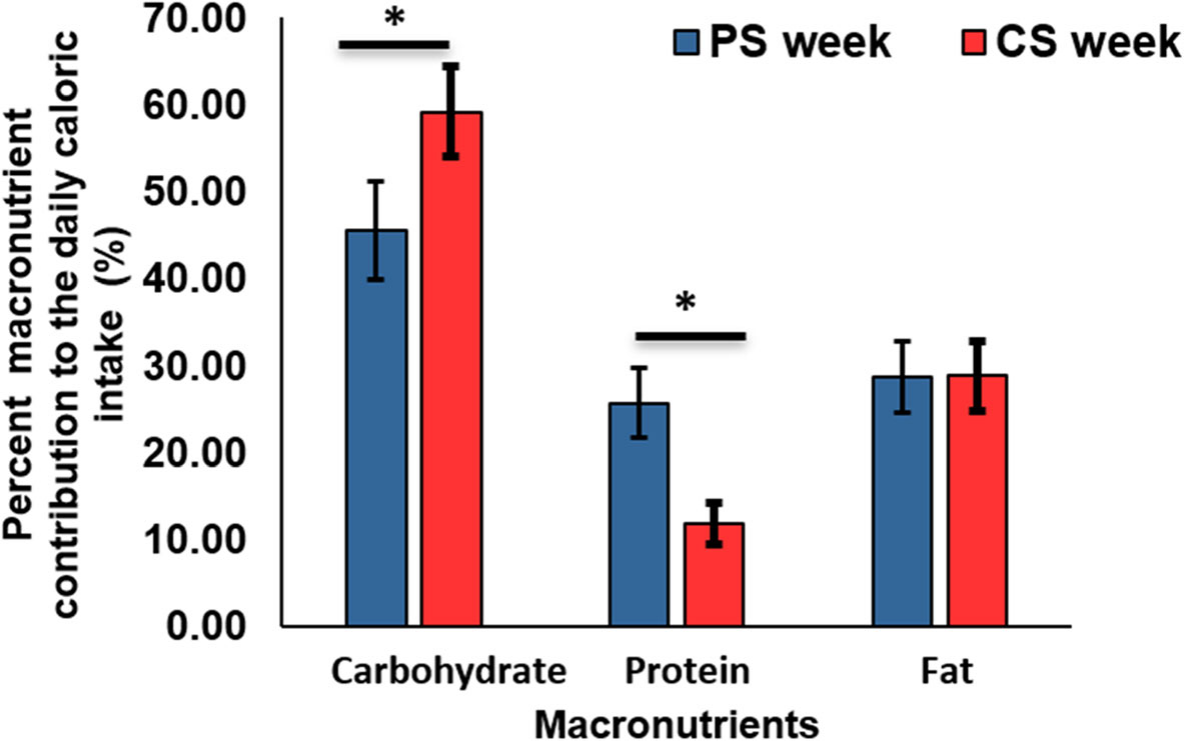
Percent contribution of macronutrients to total daily caloric intake according to treatment period (*n* = 43). PS, Protein shake; CS, Carbohydrate shake. *p value < 0.05

For the post-hoc analysis on macronutrient content, treatment order significantly impacted both the carbohydrate (*p* = 0.003) and protein models (*p* = 0.012). Therefore, we adjusted for treatment order in our model. Ad libitum protein and carbohydrate intake did not differ between treatment periods (*p* = 0.979 and *p* = 0.092, respectively). We also examined macronutrient intake as a percent of daily caloric intake (Fig. 5) The percent of total daily calories from protein during PS was significantly higher than during CS (25.75% (SD = 4.08) vs 11.88% (SD = 2.37), t = 22.11, *p* < 0.001). Similarly, the percent of total daily calories from carbohydrate during CS was significantly higher than during PS (59.27% (SD = 5.23) vs 45.54% (SD = 5.61), (t = 19.89, *p* < 0.001). However, the percent of total daily calories from fat did not differ between PS and CS and contributed about 29% of total daily calories (t = − 0.310, *p* = 0.758).

## Discussion

Our study compared the impact of a carbohydrate preload to an egg protein preload on ad libitum energy balance. The novelty of this study is that we examined the effects of carbohydrate relative to protein on energy balance over several days, without a high level of control over the diet that eliminates one macronutrient and replaces it with increased intake of the other. We studied the effects of egg protein or carbohydrate preloading on energy balance, hunger, satiety, and TEF. Furthermore, we investigated the effects of prescribed protein intake on ad libitum protein intake and prescribed carbohydrate intake on ad libitum carbohydrate intake to identify if the macronutrient intake was being physiologically regulated. Finally, we studied the percent contribution of each macronutrient to total energy intake during treatment periods to understand if supplementation of proteins or carbohydrates could increase the percent contribution of those macronutrients to energy intake in an otherwise free-choice, ad libitum setting.

We found little evidence to suggest that preloading egg protein or carbohydrate before each daily major meal, in a dose that corresponds to approximately 20% of energy needs, has differential effects on energy balance over a 5-day period. The thermic effect of the protein shake was higher than the carbohydrate shake, as expected, but this was the only indication that protein may have any differential effect on energy balance than carbohydrate. Two other relevant studies have been conducted that manipulated macronutrients and allowed ad libitum feeding. Baer and colleagues fed 56 g/day of whey, soy, or maltodextrin for 23 weeks in participants with overweight and obesity, and found that whey supplementation decreased fat mass by 1.8 kg and increased lean mass by 2.3 kg relative to maltodextrin treatment [14]. Our study was too short in duration to expect or measure body composition changes, however, Baer et al.’s results suggest that the small non-significant shifts in energy balance we detected could lead to significant differences in body composition in a longer-term study. Johnstone and colleagues overfed participants (by 0.6*RMR) on a high protein, high carbohydrate, or high fat diet for 1 day, and tracked their compensatory response for the overfeeding on the following day [15]. Although the high protein diet produced greater satiety, there was no difference between the conditions in subsequent reduction in ad libitum intake [15]. According to past literature, shifting the overall diet from 15 to 25% protein does not impact energy intake over a 4-day period [12]. However, the effect of high protein intake on reducing energy intake is evident when comparing a diet that is 15% protein to a diet that is 30% protein [10]. Hence, another possibility is that our prescribed protein supplementation was not high enough to initiate self-protective mechanisms to reduce ad libitum protein intake.

Interestingly, in our study, there was a significant treatment effect for individuals on the high dose in the secondary analysis only, such that they had higher energy balances on CS compared to PS (+ 882 kcal/wk., *p* = 0.030). This significance level was found in only 10 subjects receiving this dose, which equated to 99 g of either protein or carbohydrate per day. The doses were designed to equate to 20% of total energy needs and were therefore based on basal metabolic rate. This effect could also be attributable to effects that are specific to larger body size (which dictated the higher metabolic rate and larger dose). Given the number of tests we conducted, it is also plausible that this is simply a type I error. However, it may be that the absolute amount of protein needs to be very high to see any impact on energy balance, and that protein doses should be considered in absolute gram amounts rather than prescribed as a percent of energy needs. Nonetheless, it should be recognized that even though energy balance was more favorable in the high dose protein condition, energy balance was still positive and our findings do not support the idea that protein would help with weight maintenance in similar settings. Future studies might test the impact of this higher dose on ad libitum energy balance at a wide range of body weights.

Our findings do not support the idea that adding carbohydrate-containing foods to the diet would lead to positive energy balance more so than adding protein. This is in contrast with the carbohydrate-insulin hypothesis, which suggests the influence of carbohydrate on insulin would have a causative role in energy storage, appetite regulation, and positive energy balance. Our results suggest adding protein to the diet is essentially equivalent to adding carbohydrate to the diet, and the positive energy balance that occurred in this ad libitum buffet condition was independent of the macronutrient added to the diet. However, this does not preclude the possibility that a longer-term study or a study altering diet composition in different ways might result in body composition differences between groups, as has been reported previously with whey protein supplementation compared to carbohydrate supplementation [14].

We powered this study to have 80% power to detect a standardized mean difference between treatments of 0.57, which is considered a medium effect size [31]. It is possible that the effect size of protein compared to carbohydrate is actually smaller than 0.57. In fact, the standardized mean difference between treatments for 5-day net energy balance, overall in our trial, was 0.45 (95% CI: 0.35, 0.56). This may be the first reported effect size for this comparison and has value for informing future trials regarding the role of protein vs. carbohydrate in regulating energy balance.

Interestingly, the effect size of treatment was larger within weight status groups (SMD = + 1.045 for normal weight, − 0.748 for overweight) than the overall effect size, and the effects on each weight status were in opposite directions. The interaction was not significant, but the trial was not powered to detect this interaction. There is impairment of satiety cues in individuals with overweight or obesity, such as low production of satiety hormone PYY [32], relative to subjects with normal weight. Therefore, if egg albumin relies on those satiety cues to influence energy intake, it would be logical that effects may differ by weight status. Therefore, future studies powered to detect such an interaction between protein and weight status may be warranted.

We did not find evidence of increased satiety during the egg protein treatment through VAS measurements in normal weight individuals. Previous studies finding that egg protein is more satiating used a whole-egg breakfast paradigm and demonstrated enhanced satiety and reduced intake at the following lunch meal or the following 24 h [19, 33]. Therefore, there may be dietary components in the eggs that were not present in the egg albumin shake that are necessary for any effect. This is also supported by the fact that our findings are in line with two previous studies that examined egg albumin supplements. One demonstrated that a 20 g egg albumin preload did not differ in satiety from a 20 g preload of maltodextrin [21], while another demonstrated that a 45 g preload of egg albumen did not suppress subsequent food intake relative to a water control [20]. In addition, our protocol was less controlled than previous satiety measurement protocols. We allowed ad libitum feeding of a wide range of foods in the test meal and did not standardize the amount of food consumed in the prior morning meal when measuring satiety rather than the typical method of measuring with a standardized, single-dish meal following a standardized breakfast or morning fasting [21].

We also observed that prescribed intake of protein and carbohydrate did not affect ad libitum intake of protein and carbohydrate, respectively. Our findings are in contrast to the previously described hypothesis that protein intake is tightly regulated in the post-absorptive period [8]. Macronutrient intake regulation may be affected by an individual’s physiological and environmental characteristics [5]. One such characteristic could be acclimatization to high dietary protein intake: some populations in the northern-latitudes consume 25% of daily calories from protein [8]. However, we did not collect baseline dietary information to examine this possibility. According to the flavor-nutrient learning theory, flavor influences food choices and meal patterns possibly via peripheral nutrient sensing, central reward circuitry and gut-brain communication [34]. The carbohydrate and protein were offered as shakes, rather than their typical food forms, which may have prevented or confused established flavor-nutrient learning paradigms. Additionally, the length of time required to establish new flavor-nutrient learning mechanisms for a new foods such as the supplemental shakes are unclear; our protocol may not have provided adequate time. Furthermore, awareness of food content may affect food intake; for example, in a previous study, participants consumed more snacks after consuming a high-sugar protein shake they believed was healthy [35]. Our participants were unaware of the nutritional content of the preload shakes and thus potentially lacked this self-regulation. Taken together, this suggests that flavor and perceptions of protein-containing foods may be critical to their impact on energy intake.

According to our findings, prescribing approximately 20% of calories as a protein preload increased the total calorie contribution from protein to ~ 26%. Since there is no compensatory reduction of ad libitum protein intake by supplementation in our group of participants, it seems that it is possible to increase the protein intake by provision of protein supplements in comparison to carbohydrate supplements. Hence, protein supplementation may be a useful strategy to increase the protein intake in a person when requirement is high such as in elderly and in athletic populations.

This study has several strengths. First, a common limitation of macronutrient studies is that altering the concentration of one macronutrient in the diet experimentally is often done by eliminating or controlling the concentration of another to keep energy intake constant. While preload macronutrients were prescribed in this study, macronutrients and energy intake from the buffet and snack periods were ad libitum. Second, preload caloric doses were based on BMR to ensure that variations in energy metabolism were not due to inappropriate doses. Third, energy intake during meals was measured objectively. The cross-over design also minimized the potential for between subject confounding. Finally, participants were blinded to shake nutritional content.

Several limitations should also be considered. First, the 5-day treatment period may not have been sufficient to detect a net change in energy balance. However, a short-term study was the logical antecedent to a longer one. Second, buffet-style meals have been shown to increase food intake relative to typical, lower variety meal time conditions [24]. This may have masked any effects that would occur in ad libitum settings. Third, we did not prescribe or record whether participants chose to eat as a group or sit individually. Nor did we place time restrictions on shake consumption, time between shake consumption and buffet consumption, or time allotted for buffet consumption. These factors may have impacted eating behaviors and future studies may document or prescribe these conditions. Fourth, there was a distinct lack of sensory cues that typically come with consumption of protein or carbohydrate-rich foods, which may influence the level of satiety one experiences when consuming a food [36]. The absence of these cues limits the generalizability of our findings. Although we did not distinguish between meal-liking for the shakes vs. buffet food, Tey et al. found that liquid preload energy density predicted subsequent energy compensation more than taste quality [36]. We did not systematically probe about perceived differences in the shakes during the debriefing. Another consideration is the influence of egg protein on GI health. However, meal-liking was not influenced by the presence of GI symptoms, and energy intake was higher in those with GI symptoms compared to those without them consistently in both conditions. Therefore, it is unlikely the symptoms influenced treatment effects. Additionally, tri-axial accelerometers do not perfectly detect non-ambulatory movement (e.g. biking, weight training, etc.) when worn at the waist, and they are not waterproof (i.e. not worn while swimming). Thus, our estimates of PAEE are imperfect and plausibly underestimated. We asked participants to complete a physical activity log in order to account for activities that may have been missed, but participants were deemed to have unreliably done so and this information was not used in this analysis. Also, we did not measure 24-h nitrogen excretion as a biomarker of protein intake. If we had, we could have examined if our protein preloads were in excess of individual requirements. We also did not monitor or match female participants according to menstrual cycle, an omission that may have contributed to the variability of our results. Finally, our results can only be applied to the compensatory response to be expected from maltodextrin or egg white protein preloads.

## Conclusions

Our study found no significant differences between a protein preload and a carbohydrate preload on energy balance. Future long-term studies should examine higher doses of egg albumin, and verify whether small, non-significant effects might eventually accumulate into sustained differences between egg protein and maltodextrin in energy balance and body composition.

## Supplementary information

**Additional file 1.** Snack and Buffet Menus. listing of food items offered as snacks and buffet meals.

**Additional file 2.** CONSORT 2010 checklist of information to include when reporting a randomised trial.

## Data Availability

The datasets generated and analyzed during the current study are available in the openICPSR repository, [PERSISTENT WEB LINK TO DATASETS WILL BE FILLED IN UPON ACCEPTANCE FOR PUBLICATION].

## Abbreviations

BMI: Body mass index
BMR: Basal metabolic rate
CS: Carbohydrate shake
EB: Energy balance
EI: Energy expenditure
GID: Gastrointestinal discomfort
PAEE: Physical activity energy expenditure
PS: Protein shake
RMR: Resting metabolic rate
TEE: Total energy expenditure
TEF: Thermic effect of food
VAS: Visual analogue scale
