# Supplementary material for: A randomized cross-over trial to determine the effect of a protein vs. carbohydrate preload on energy balance in ad libitum settings
Source: Nutr J. 2019 Nov 9;18:69. doi: 10.1186/s12937-019-0497-4 (PMC6842484; doi:10.1186/s12937-019-0497-4)
Supplement: Supplementary file 1 — Additional file 1. Snack and Buffet Menus. listing of food items offered as snacks and buffet meals. [file 12937_2019_497_MOESM1_ESM.pdf]

**Day 1**				
**Meal/Food**				
**Breakfast**				
Shake (A/B) drink before meal				
Bagel				
Cream cheese, regular				
Jelly, regular				
Turkey sausage				
Banana				
Beverage:				
**Lunch**				
Shake (A/B) drink before meal				
White bread				
Deli turkey				
Kraft singles cheese				
Mayonnaise, regular				
Mustard				
Pretzels				
Orange				
Beverage:				
**Dinner**				
Shake (A/B) drink before meal				
Chicken breast				
White rice				
Broccoli				
White roll				
Beverage:				

**Day 2**				
**Meal/Food**				
**Breakfast**				
Shake (A/B) drink before meal				
Boiled eggs				
English muffin				
Butter				
Jelly, regular				
Turkey bacon				
Grapes				
Beverage:				
**Lunch**				
Shake (A/B) drink before meal				
Hamburger patty				
Hamburger bun				
Kraft 2% singles cheese				
Ketchup				
Mustard				
Kraft light mayonnaise				
Baked Lays chips				
Quaker chewy granola bar				
Canned peaches, heavy syrup				
Beverage:				
**Dinner**				
Shake (A/B) drink before meal				
Tilapia				
English peas, canned				
Carrots, canned				
White roll				
Butter				
Jello, regular				
Beverage:				

**Day 3**				
**Meal/Food**				
**Breakfast**				
Shake (A/B) drink before meal				
Pancakes				
Pancake syrup				
Promise margarine				
Meatless sausage links				
Strawberries, frozen				
Beverage:				
**Lunch**				
Shake (A/B) drink before meal				
Hoagie roll				
Chicken salad				
Sunchips				
Mandarin oranges, in juice				
Rice Krispie treat				
Beverage:				
**Dinner**				
Shake (A/B) drink before meal				
Pork tenderloin				
Sweet potato				
Butter				
Brown sugar				
Spinach, canned				
Crescent rolls				
Beverage:				

**Day 4**				
**Meal/Food**				
**Breakfast**				
Shake (A/B) drink before meal				
Boiled eggs				
Grits				
Bacon				
Butter				
Canned pears, heavy syrup				
Beverage:				
**Lunch**				
Shake (A/B) drink before meal				
White bread				
Deli ham				
Cheddar cheese slices				
Kraft real mayonnaise				
Mustard				
Vegetable soup				
Apple				
Beverage:				
**Dinner**				
Shake (A/B) drink before meal				
Meatloaf				
Ketchup				
Mashed potatoes, prepared				
Butter				
Green beans, canned				
White roll				
Oreo cookies				
Beverage:				

**Day 5**				
**Meal/Food**				
**Breakfast**				
Shake (A/B) drink before meal				
Oatmeal, plain				
Blueberries, frozen				
Sugar				
Butter				
Turkey sausage				
Beverage:				
**Lunch**				
Shake (A/B) drink before meal				
Sub roll				
Meatballs				
Marinara sauce				
Doritos				
Mixed fruit, 100 calorie				
Beverage:				
**Dinner**				
Shake (A/B) drink before meal				
Turkey breast, baked				
Macaroni and cheese				
Lettuce				
Kraft Italian dressing				
Tomato, chopped				
Cucumber, chopped				
Breadsticks				
Pineapple, juice packed				
Chocolate pudding, fat-free				
Beverage:				

Cohort # 1 snacks

**Snack**	
Apple	
Mandarin oranges	
Banana	
Peanuts, salted	
Peanut butter	
Saltine crackers	
Graham crackers	
Regular Lay's chips	
Baked Lay's chips	
Fudge stripe cookies	
Teddy Grahams	

**Day 1**				
**Meal/Food**				
**Breakfast**				
Shake (A/B) drink before meal				
Bagel				
Cream cheese, regular				
Jelly, regular				
Turkey sausage				
Banana				
Beverage:				
**Lunch**				
Shake (A/B) drink before meal				
White bread				
Deli turkey				
Kraft singles cheese				
Mayonnaise, regular				
Mustard				
Pretzels				
Brownie				
Beverage:				
**Dinner**				
Shake (A/B) drink before meal				
Chicken breast				
Macaroni and cheese				
Broccoli				
White roll				
Cherry mixed fruit				
Beverage:				

**Day 2**				
**Meal/Food**				
**Breakfast**				
Shake (A/B) drink before meal				
Boiled eggs				
English muffin				
Butter				
Jelly, regular				
Turkey bacon				
Mandarin oranges				
Beverage:				
**Lunch**				
Shake (A/B) drink before meal				
Hamburger patty				
Hamburger bun				
Kraft 2% singles cheese				
Ketchup				
Mustard				
Kraft light mayonnaise				
BBQ potato chips				
Canned pears				
Beverage:				
Dinner				
**Shake (A/B) drink before meal**				
Tilapia				
Tartar sauce				
English peas, frozen				
Baked potato				
White roll				
Butter				
Sour cream				
Chocolate chips ahoy cookie				
Beverage:				

**Day 3**				
**Meal/Food**				
**Breakfast**				
Shake (A/B) drink before meal				
Pancakes				
Pancake syrup				
Promise margarine				
Pork sausage pattty				
Blueberries				
Beverage:				
**Lunch**				
Shake (A/B) drink before meal				
Croissant				
Chicken salad				
Doritos				
Diced peaches				
Rice Krispie treat				
Beverage:				
**Dinner**				
Shake (A/B) drink before meal				
Pork roast				
White rice				
Butter				
Caribbean vegetables				
Crescent rolls				
Edwards lemon pie				
Beverage:				

**Day 4**				
**Meal/Food**				
**Breakfast**				
Shake (A/B) drink before meal				
Boiled eggs				
Grits				
Bacon				
Butter				
Biscuit, frozen				
Jelly				
Beverage:				
**Lunch**				
Shake (A/B) drink before meal				
Whole wheat bread				
Deli ham				
Cheddar cheese slices				
Kraft real mayonnaise				
Mustard				
Regular potato chips				
Pound cake				
Beverage:				
**Dinner**				
Shake (A/B) drink before meal				
Meatloaf				
Ketchup				
Mashed potatoes, prepared				
Butter				
Green beans, canned				
White roll				
Oreo cookies				
Pineapple				
Beverage:				

**Day 5**				
**Meal/Food**				
**Breakfast**				
Shake (A/B) drink before meal				
Oatmeal, plain				
Brown sugar				
Blueberries, frozen				
Butter				
Turkey sausage				
Beverage:				
**Lunch**				
Shake (A/B) drink before meal				
Sub roll				
Meatballs				
Marinara sauce				
Doritos				
Applesauce				
Beverage:				
**Dinner**				
Shake (A/B) drink before meal				
Turkey breast, baked				
Sweet potato				
Brown sugar				
Butter				
Lettuce				
Kraft Italian dressing				
Tomato, chopped				
Cucumber, chopped				
Breadsticks				
Chocolate pudding, fat-free				
Beverage:				

All other cohorts- snacks

**Snack**	
Granola bar (avg)	
Banana	
Peanut butter	
Graham crackers	
Chips (avg)	
Baked Lay's chips	
Lorna Doone cookies	
Swiss cake roll	
Skim milk	
Yogurt	
Cereal (avg)	
Oreos	
Animal crackers	
Pear, fresh	
Famous Amos cookies	
Quaker granola bar	
Nature Valley granola bar	
Saltine crackers	
Ranch dressing	
**Beverage:**	
Skim milk	
Whole milk	
Apple juice	
Orange juice	
Coke	
Sprite	
Mt Dew	
Dr. Pepper	
Gingerale	
Diet drinks	
